# High Fat High Sugar Diet Reduces Voluntary Wheel Running in Mice Independent of Sex Hormone Involvement

**DOI:** 10.3389/fphys.2017.00628

**Published:** 2017-08-25

**Authors:** Heather L. Vellers, Ayland C. Letsinger, Nicholas R. Walker, Jorge Z. Granados, J. Timothy Lightfoot

**Affiliations:** Department of Health and Kinesiology, Texas A&M University College Station, TX, United States

**Keywords:** diet, wheel running, mice, sex hormones, chronic overfeeding

## Abstract

**Introduction:** Indirect results in humans suggest that chronic overfeeding decreases physical activity with few suggestions regarding what mechanism(s) may link overfeeding and decreased activity. The primary sex hormones are known regulators of activity and there are reports that chronic overfeeding alters sex hormone levels. Thepurpose of this study was to determine if chronic overfeeding altered wheel running through altered sex hormone levels.

**Materials and Methods:** C57BL/6J mice were bred and the pups were weaned at 3-weeks of age and randomly assigned to either a control (CFD) or high fat/high sugar (HFHS) diet for 9–11 weeks depending on activity analysis. Nutritional intake, body composition, sex hormone levels, and 3-day and 2-week wheel-running activity were measured. Additionally, groups of HFHS animals were supplemented with testosterone (males) and 17β-estradiol (females) to determine if sex hormone augmentation altered diet-induced changes in activity.

**Results:** 117 mice (56♂, 61♀) were analyzed. The HFHS mice consumed significantly more calories per day than CFD mice (male: *p* < 0.0001; female: *p* < 0.0001) and had significantly higher body fat (male: *p* < 0.0001; female: *p* < 0.0001). The HFHS diet did not reduce sex hormone levels, but did significantly reduce acute running-wheel distance in male (*p* = 0.05, 70 ± 28%) and female mice (*p* = 0.02, 57 ± 26%). In animals that received hormone supplementation, there was no significant effect on activity levels. Two-weeks of wheel access was not sufficient to alter HFHS-induced reductions in activity or increases in body fat.

**Conclusion:** Chronic overfeeding reduces wheel running, but is independent of the primary sex hormones.

## Introduction

Physical inactivity is a major worldwide concern that has led to a host of non-communicable diseases including obesity, heart disease, type II diabetes, some forms of cancer, and even decreased life-expectancy (Lee et al., 2012). Lee et al. (2012) examined world-wide physical inactivity prevalence and estimated that out of 57 million deaths reported in 2008, that 5.3 million of the deaths (9%) were due to physical inactivity. In the same study, the authors further demonstrated from the estimated deaths due to physical inactivity, that if physical activity were increased by 10–25%, that approximately 1.3 million of the reported deaths could have been averted (Lee et al., 2012). More recently, a study by Ding et al. (2016) estimated that physical inactivity itself has led to a world-wide economic burden of $53.8 billion in yearly health care costs combined with an additional $13.7 billion costs related to productivity losses in 2013. While an increase in physical activity by even a modest amount (~1,000 total calorie expenditure per week) has been shown to significantly decrease all-cause mortality (Lee and Skerrett, 2001) and lower premature death (Bowen et al., 2011), activity levels still remain low and continue to decline. It is commonly believed that physical activity participation is largely determined by the “built environment”—the presence and ease of access to sidewalks, parks, trails, recreational facilities, etc.—however, studies which have objectively measured activity in individuals surrounded by such environments, do not support this association. To date, it remains unclear what factors contribute to the continual decline in daily activity for the individual, and thus, raises the need for scientific research to not only identify factors inhibiting physical activity, but also the mechanistic pathway(s) regulating an individual's participation in activity.

Copious literature shows that physical activity is regulated by genetic/biological mechanisms (25–92% influence; Lightfoot, 2011), and to date, the primary sex hormones in males (testosterone) and females (17-β estradiol) are known as the most potent biological regulators of voluntary wheel running in mice (Wang et al., 1925; Bowen et al., 2011, 2012). For example, a prior study from our lab demonstrated that removal of testosterone or 17β-estradiol in mice (via orchiectomy or ovariectomy) resulted in approximately 90% decreased daily activity in mice, while endogenous replacement of these hormones resulted in varying levels (35–110%) of recovered baseline activity (Bowen et al., 2012). While the biological regulation of physical activity is strongly influenced by the primary sex hormones, few studies have tested whether a factor(s) known to disrupt the levels of the sex hormones could have a direct effect on activity levels. To date, the only study to our knowledge which has tested such a link, is a recent study from our lab by Schmitt et al. (2016), which demonstrated that disruption to the levels of testosterone and 17β-estradiol by a common environmental endocrine disruptor benzylbutylphthalate (BBP), decreased wheel running in mice. Thus, physical activity can be inhibited by exposure(s) that disrupt the levels of the primary sex hormones.

Chronic overfeeding, defined here as excess caloric intake above weight maintenance needs, has been shown to directly alter the levels of the primary sex hormones (Bouchard et al., 2014), and in separate sources of literature, has also been indirectly associated with reductions in physical activity in humans (Levine et al., 2008; Schmidt et al., 2012). Levine et al. (2008) showed that 8 weeks of overfeeding in both lean and obese human subjects by 1,000 kcal/day, decreased their daily walking distance. In another study, Schmidt et al. (2012) showed that following a shorter duration (3 days) of overfeeding, a caloric intake of 1.4 times greater than individual basal metabolic needs led to reduced spontaneous physical activity for the obesity prone individual. However, no study has directly tested, or proposed, specific mechanisms mediating this response.

The primary purpose of this study was to determine if chronic overfeeding altered acute wheel running in mice, and then whether the primary sex hormones were associated with any potential alteration in wheel running. Secondarily, an additional experiment was devised to determine whether providing long-term access to a running wheel would alter diet-induced alterations in activity. We hypothesized that chronic overfeeding would significantly reduce the sex hormone levels in male (testosterone) and female (17β-estradiol) mice, and that exogenous sex hormone supplementation would demonstrate the sex hormones and mediators between overfeeding and reduced wheel running activity. Determining whether a causal link between chronic overfeeding and physical inactivity exists would support the hypothesis that environmental factors can directly influence the biological mechanisms controlling daily activity as recently suggested by Schmitt et al. (2016).

## Materials and methods

### Animals

This protocol conformed to the standards of humane animal care and was approved by the Texas A&M University Institutional Animal Care and Use Committee (AUP 2013-0274). All animals were housed in the University Vivarium with 12-h light/dark cycles. Inbred C57BL/6J breeder mice (Jackson Laboratory, Bar Harbor, ME) were used for all three experiments (listed below) because of their consistent use in the scientific literature and their genetic homogeneity.

Three sets of experiments were conducted to fulfill the purpose of this project. In all three experiments, mice were developed using four triads of C57BL/6J mice (4♂, 8♀; two females were matched with each male). At 3 weeks of age, pups were weaned, and individually housed using random assignment to one of two diets: a control diet (CFD) or a high fat/high sugar (HFHS) diet (see below for diet compositions). Mice were assigned to one of the groups listed in Table 1, with the goal of each group having a target of six of each sex per group. Further, throughout all experiments, all females were sacrificed when they were in the Proestrus stage of the estrous cycle (see below). Additionally, to eliminate circadian cycle influences on sex hormone levels, all male mice were sacrificed between 9:00–11:00 am, and the female mice were sacrificed between 12:00–2:00 pm during the light phase (i.e., inactive phase) of the light-dark cycle as in the study by McLean et al. (2012) (time frame during Proestrus with the highest peak of estrogen; McLean et al., 2012).

**Table 1:** Summary of mouse random group assignments.

**Experimental aim**	**Wheel access (days)**	**Group**	**No. males (*n* = 56)**	**No. females (*n* = 61)**
1	0	CFD	6	6
	0	HFHS	5	6
	3	CFD+wheel	5	7
	3	HFHS+wheel	6	6
				
2	0	HFHS+T	6	–
	0	HFHS+E2	–	5
	3	CFD+T+wheel	6	–
	3	HFHS+T+wheel	6	–
	3	CFD+E2+wheel	–	5
	3	HFHS+E2+wheel	–	7
	3	HFHS sham	6	5
				
3	14	CFD+wheel	5	7
	14	HFHS+wheel	5	7

*CFD, Control diet; HFHS, high fat/high sugar; CFD+wheel, control diet with two-week wheel access; HFHS+wheel, high fat high sugar diet with 2 week wheel access; HFHS+T, high fat high sugar diet with testosterone supplementation; HFHS+E2, high fat high sugar diet with 17β-estradiol supplementation; CFD+T+wheel, control diet with testosterone supplementation and wheel access; HFHS+T+wheel, high fat high sugar diet with testosterone supplementation and wheel access; CFD+E2+wheel, control diet with 17β-estradiol supplementation and wheel access; HFHS+E2+wheel, high fat high sugar diet with 17β-estradiol supplementation with wheel access; HFHS+Sham, high fat high sugar diet with sham surgical implantation*.

### Experiment 1 overview

This experiment was designed to determine if chronic overfeeding, via the HFHS diet, altered daily wheel running and/or sex hormone levels. After weaning, the animals were randomly assigned a diet from 3 weeks to 12 weeks of age, with the amount of kcal consumed determined weekly. Following 9 weeks of overfeeding (i.e., mice were 12 weeks of age), and just prior to receiving any other form of treatment (e.g., running-wheel), body composition measurements (see below) were compared between all of the CFD and HFHS mice. Also at 12 weeks of age, animals in both diet groups were randomly assigned to a group with or without running-wheel access. The mice that did not receive running-wheels were sacrificed to determine the effect of the HFHS diet on sex hormone levels. For the groups that received a running-wheel (see below), the first 2 days of running-wheel access was designated as an acclimation period, while day 3 of running-wheel activity was measured and used in the analysis. Acute wheel activity was measured to remove the potential training effects of longer time periods of wheel running (Dawes et al., 2015). Weight and body composition were determined weekly. All animals were then sacrificed via inhalation of 3–4% isoflurane with subsequent cervical dislocation, with serum harvested for later analysis of sex hormones.

### Dietary protocols

The mice were randomly assigned to of one two diets: a high fat/high sugar diet (HFHS) or control diet (CFD). The HFHS diet combined the high fat diet from Research Diets, Inc. [product D12451, New Brunswick, NJ; 20% protein, 35% carbohydrate (sucrose), and 45% fat (6% soybean oil, 39% lard)] with a 20% fructose solution to replace normal water. For the control diet, a normal 4% fat chow diet (Harlan Labs, Houston TX; 25.2% protein, 39.5% carbohydrate, 3.3% crude fiber, 10% neutral fiber, and 9.9% ash), was used in conjunction with water. Food consumption (grams) and fluid intake (ml) were weighed and recorded on a weekly basis to estimate the average daily/weekly caloric intake.

### Estrous cycle determination

Because sex hormone levels fluctuate throughout the estrous cycle in female mice every 3–4 days, the vaginal lavage technique provided by McLean et al. (2012) was used to determine estrous cycle phase. Prior to performing vaginal lavages, phosphate-buffered saline (PBS) was autoclaved and cooled to physiological temperature (i.e., 37°C). Approximately 25–50 μl of the PBS was displaced into the opening of the vaginal canal and pipetted 4–5 times to ensure a sufficient number of cells were taken, and the sample was smeared on a glass slide, then allowed to completely dry at room temperature before analysis. Once the estrous smears dried, the slides were then stained using the three-step Hema 3 Fixative and Solutions containing fixative, hematoxylin, eosin, and four deionized waters (for destaining). The slides were then viewed at 10X under a microscope. The estrous cycle phases were determined by cell typology using the guidelines provided by McLean et al. (2012).

### Wheel-running activity

Wheel running activity was measured using our validated and repeatable standard protocol (Lightfoot et al., 2004; Turner et al., 2005). Briefly, solid surface running-wheels (Kaytee®, Chilton, WI) with a 410 mm circumference were mounted to the cage tops of standard rat cages. The magnet from a cycling computer (BC8.12, Sigma Sport, Batavia, IL) was glued to the wheel and the cycling computer was attached to the outside of the cage and calibrated to the size of the wheel. Running distance (km/day) and duration (mins/day) data were collected every 24-h, and the average daily running speed (m/min) was calculated from the daily distance and duration measures. The sensor alignment and freeness of the wheel were checked daily and adjusted as needed.

### Body composition

Weight and body composition were determined using an Echo MRI mouse body composition device (Houston, TX). Body composition measurements—weight, body fat percentage, and lean tissue weights—were determined by placing the mouse in the appropriate analysis tube of the MRI machine, and inserting into the MRI. Each measurement took approximately 60 s.

### Sex steroid determination

At sacrifice, blood samples were collected via a cardiac puncture using a 20-gauge needle, and then each blood sample remained at room temperature for 30–60 min or until a clear separation between the blood and sera began to occur. All samples were then centrifuged at 10,000 rpm for 30 min at 4°C to separate red blood cells and serum. The serum samples were aliquoted into separate tubes and then stored in an −80°C freezer for triplicate assessment of testosterone and 17β- estradiol using competitive ELISA techniques (Alpco Serum Testosterone, 55-TESMS-E01, Salem, NH; Abcam Estrogen Kit, ab108667, Cambridge, MA). In the female mice, we also collected uterine horn weights, which is considered an indirect indicator of biologically active estrogens (Wang et al., 2005). On the day of sacrifice, the left and right uteri horns of the female mice were dissected, adhering fat and mesentery tissue were removed, and the uterine horns then weighed on an electronic scale (Mettler Toledo, Columbus, OH). Uterine horn weights were standardized by calculating the ratio of the uterine wet weight (mg) and total body weight of each mouse (mg).

### Experiment 2 overview

The purpose of this experiment was to determine whether exogenous sex hormone supplementation following overfeeding, would alter the acute HFHS diet effect on wheel running activity in mice. The mice in this experiment were produced and monitored in a similar fashion as described in Experiment 1. At 12 weeks of age, all mice in this experiment received a silastic capsule implant (see below) that was either empty (sham) or filled with an endogenous sex hormone depending on the sex of the animal (females: 17β-estradiol; males: testosterone). Following 5 days of recovery, mice were sacrificed (without having wheel access; Table 1) and hormone levels were determined using methods from Experiment 1 to determine efficacy of the implants. The remaining mice received a running-wheel and acute activity was measured (as described in Experiment 1) to determine if endogenous supplementation affected wheel running levels. After activity measurements (at 13.5 weeks old), the mice were sacrificed and tissues harvested as described in Experiment 1.

### Endogenous testosterone and 17β-estradiol implant

The silastic implant procedures were performed in a similar fashion as described in previous work from our lab (Bowen et al., 2011). Briefly, a 10 mm section of the silastic tubing (Dow Corning, Midland, MI) with an outer diameter of 2.16 mm and inner diameter of 1.02 mm was packed with either powder testosterone or powder 17β-estradiol (Sigma-Aldrich, St. Louis, MO) and the ends of the tubing sealed with weatherproof silicone glue. Each implant was washed in 70% alcohol for 1 min, rinsed in deionized water, and then patted dry and stored in Eppendorf tubes at room temperature under dark, dry conditions. Sham capsules were made in a similar fashion without inclusion of the sex hormone. In this study, only Sham groups for HFHS fed male and female mice were implemented given our prior studies which demonstrated no effect of the silastic implant itself on wheel running behaviors in normal (CFD fed) mice (Bowen et al., 2011, 2012). At 12 weeks of age, mice were anesthetized with isoflurane and a small incision made on the caudal lateral aspect of the neck with a cavity about 15 mm in depth and width opened between the skin and muscle tissue. The silastic implant was inserted into the cavity and the incision wound was closed with a surgical clip. The mouse was allowed to recover for 5 days before further measurements were made. During the recovery days, the mice remained on the same dietary treatment that they were initially assigned.

### Experiment 3 overview

This experiment determined whether 2-weeks of running-wheel access would recover the HFHS-induced alterations to body composition and wheel-running activity. At 12 weeks of age, running-wheel access was provided for 2 weeks for all mice; during this time, all measurements (weight, body composition, activity, sex hormones) were conducted in a similar fashion as in Experiment 1. Additionally, body composition was also measured at 14 weeks of age and then compared to values at 12 weeks of age to determine if the 2-week wheel access period was sufficient to reduce percent body fat.

### Statistical analyses

In all analyses, the alpha-value was set *a priori* at 0.05. Given it is well-established that activity measures in male and female mice are significantly different, where females typically run approximately 20% farther on average than males (Lightfoot et al., 2004), we employed separate two-way ANOVAs (factors = diet, weight) to determine the effects of diet and sex hormone supplementation on acute wheel running measures. In Experiment 3, data were analyzed using a two-way ANOVA with diet as a factor and time as the repeated measure. Body composition measures (percent body fat, fat mass, lean mass, and total body weight), were analyzed by a one-way ANOVA while the nutritional intake data were analyzed by a two-way ANOVA with time being the repeated measure and the alpha level set *a priori* at 0.05. In the event of significant main effects in any analysis, a Tukey's *post-hoc* test was employed using an alpha level set at 0.05. Additionally, a one-way ANOVA within each sex to investigate reproducibility of the overfeeding-induced, acute running-wheel activity indices compared CFD and HFHS- animals between experiments 1 and 3. All statistical analyses were completed using JMP statistical software (SAS Inc., Cary, NC), while all graphs were developed using GraphPad Software (La Jolla, CA).

## Results

### Mice demographics, caloric intake and body composition

**Figure 1:**
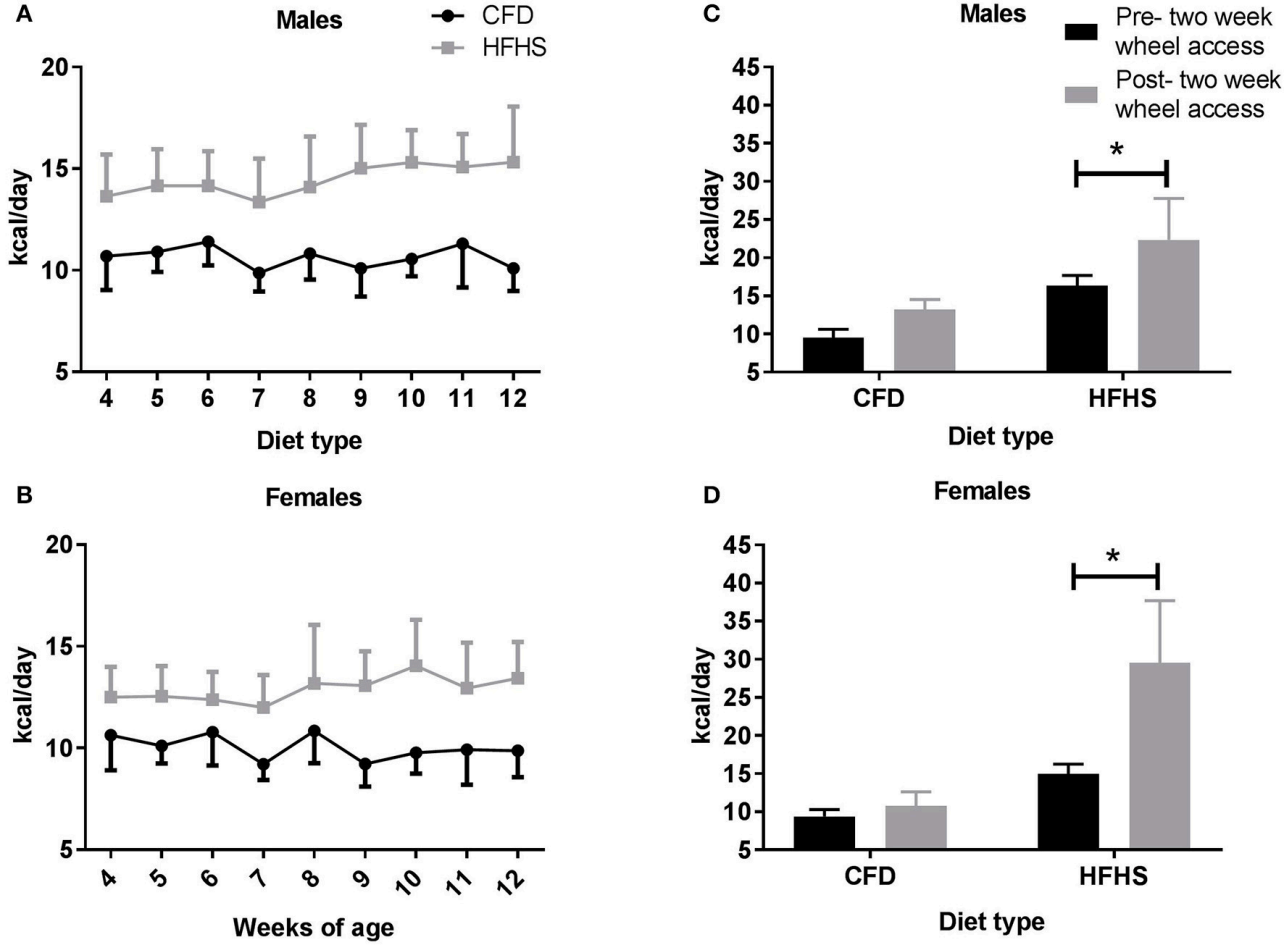
Average daily caloric (kcal) intake in all mice from 4 to 14 weeks of age. CFD, control mice; HFHS, high fat/high sugar mice. **(A)** Male mice average caloric intake across weeks of study. **(B)** Female mice average caloric intake across weeks of study. **(C)** Male average daily caloric intake pre- and post-2-week wheel access. **(D)** Female average daily caloric intake pre- and post-2-week wheel access. ^*^Significant difference in average daily caloric intake between CFD and HFHS male and female mice (*p* < 0.05).

For this study, 117 mice (56♂, 61♀, Table 1) were developed with an average litter size of seven pups (±1) in the control (CFD) mice and eight pups (±1) in the HFHS mice. From 4 to 12 weeks of age, the HFHS male mice consumed significantly more kilocalories per day (kcals/day) compared to their control counterparts (*p* < 0.0001; Figure 1A). During this period, the HFHS male and female mice consumed on average 26.3 and 22% more kcals/day than the CFD mice, respectively. For male mice that received a running wheel for 2-weeks, beginning at 12 weeks of age, the HFHS mice significantly increased their caloric consumption with wheel running access (pre- 2-week wheel running access, 16.3 kcals/day ± 1.4 vs. post- 2-week wheel running, 22.4 ± 5.4, *p* = 0.01; Figure 1C), while the CFD daily kcal intake did not change significantly (9.5 kcals/day ± 1.1 vs. 13.2 ± 1.3, *p* = 0.10; Figure 1C). Similarly, female HFHS mice consumed significantly more kcals/day per day compared to the CFD female mice across the overfeeding period (10.04 ± 0.62 vs. 12.9 ± 0.61 kcals/day; *p* < 0.0001; Figure 1B). For female mice that received a running wheel for 2-weeks, the HFHS mice significantly increased their caloric consumption with running access (pre- 2-week wheel running access, 15.0 kcals/day ± 1.3 vs. post- 2-week wheel running, 29.4 ± 5.4, *p* = 0.01; Figure 1D), while the CFD daily kcal intake did not change significantly (9.4 kcals/day ± 0.9 vs. 10.8 ± 1.8, *p* = 0.10; Figure 1D).

**Figure 2:**
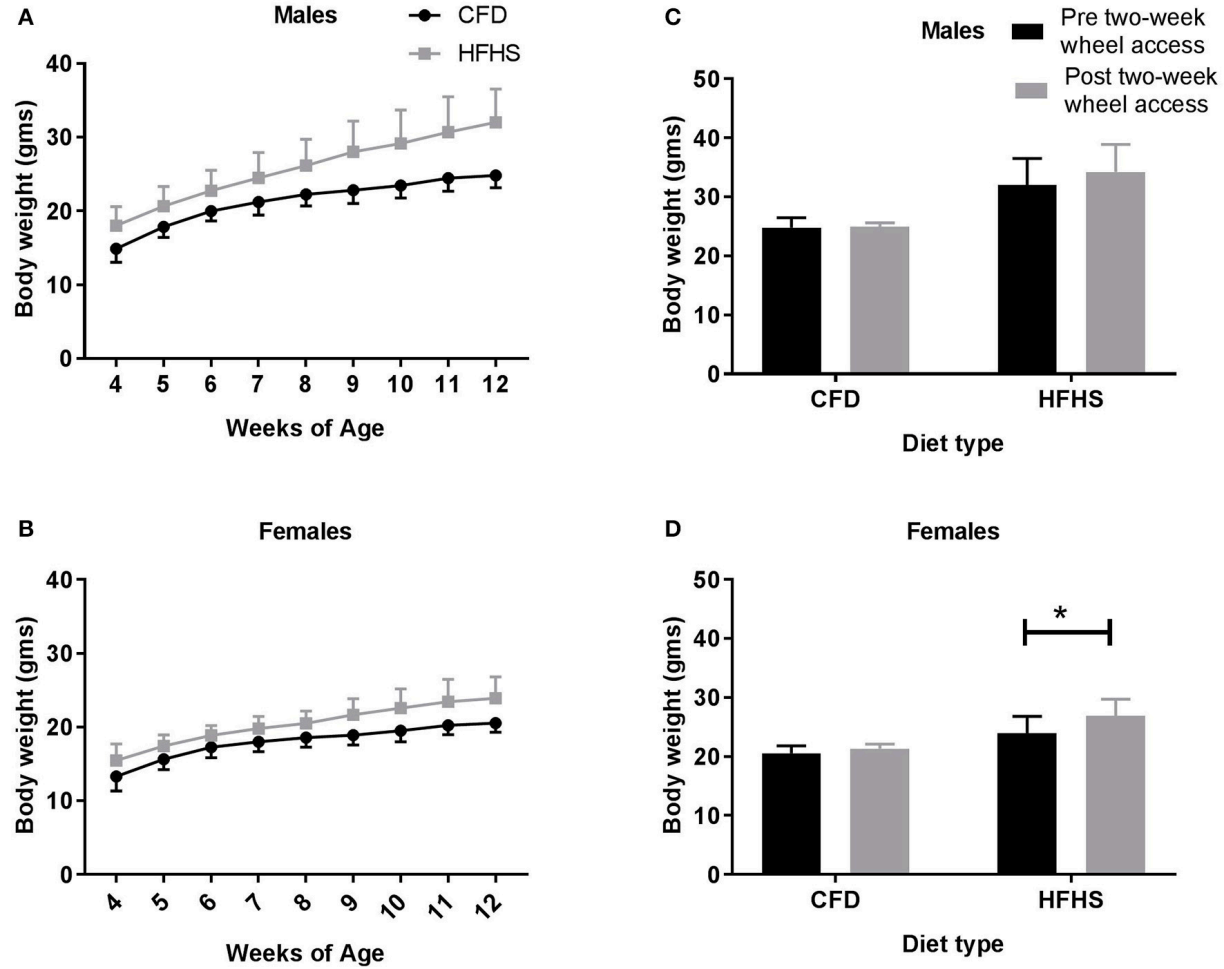
Average weekly body weight (gms) in all mice. **(A)** Male average weekly body weight (gms) from 4 to 12 weeks of age. **(B)** Female average weekly body weight (gms) from 4 to 12 weeks of age. **(C)** Male average weekly body weights pre- and post-2-week wheel access. **(D)** Female average weekly body weights pre- and post-2-week wheel access. ^*^Significant difference in the average body weights measured at 12 weeks of age (pre-2-week wheel access) and 14 weeks of age (post-2-week wheel access) in the CFD and HFHS male and female mice (*p* < 0.05).

Over time (4–12 weeks of age), the average weekly body weights were significantly higher in the HFHS fed mice when compared to CFD fed mice, in both males (*p* < 0.0001; Figure 2A) and females (*p* < 0.001; Figure 2B). When comparing all CFD and HFHS mice at 12 weeks age, the male HFHS mice groups had higher total body weight (CFD vs. HFHS: 24.9 ± 1.5 vs. 32.3 ± 3.4 g; *p* < 0.0001), body fat percentage (12.1 ± 3.5 vs. 38.4 ± 14.3%; *p* < 0.0001), fat mass (2.6 ± 0.8 vs. 8.6 ± 3.4 g; *p* < 0.0001), and lean mass (21.1 ± 09 vs. 22.2 ± 1.6 g; *p* = 0.001) than the CFD male mice. Likewise, the HFHS female mice displayed higher total body weight (CFD vs. HFHS, 20.6 ± 1.2 vs. 23.9 ± 2.8 g; *p* < 0.0001), body fat percentage (12.9 ± 3.4 vs. 25.7 ± 10.8%; *p* < 0.0001), fat mass (2.2 ± 0.6 vs. 4.6 ± 2.1 g; *p* < 0.0001), and lean mass (16.9 ± 1.0 vs. 17.8 ± 1.0 g; *p* = 0.001) than the control mice. In the last experimental aim of this study, where the mice received a running wheel for 2-weeks, a significant main effect of diet was indicated with the HFHS male (*p* < 0.0001; Figure 2C) and female (*p* < 0.0001; Figure 2D) mice weighing significantly more than their control counterparts (prior to receiving a running wheel). In males, when comparing the average body weights measured pre- and post-2-week wheel access there was not a significant difference in body weights in either the CFD (*p* = 0.99; Figure 2C) or HFHS (*p* = 0.37; Figure 2C) mice. In females, while the average body weights measured pre- and post- 2-week wheel access were not different in the CFD mice (*p* = 0.65; Figure 2D), the HFHS mice displayed significant gains in body weight even after having access to a wheel (*p* = 0.005; Figure 2D).

### Experiment 1—acute wheel-running and sex hormones

**Figure 3:**
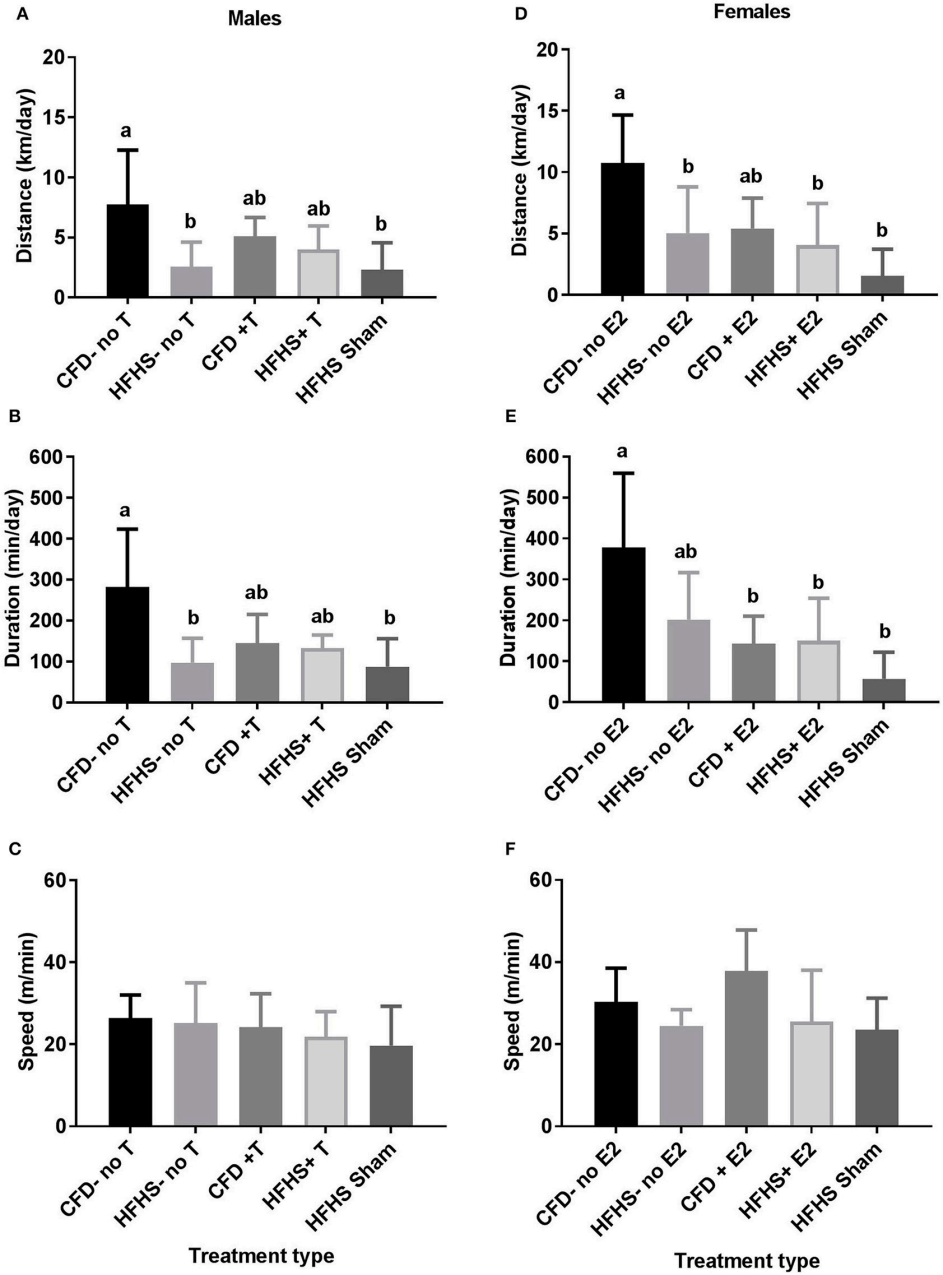
Acute running-wheel activity in CFD and HFHS male and female mice with- and without supraphysiological sex hormone supplementation. **(A–C)**: Male acute running-wheel distance, duration, and speed in response to diet and supraphysiological testosterone supplementation. **(D–F)**: Female acute running-wheel distance, duration, and speed in response to diet and supraphysiological 17β-estradiol supplementation. Bars not connected by the same letter are significantly different (*p* < 0.05). Male mice groups **(A–C)**: CFD-no T, control diet without testosterone supplementation; HFHS-no T, high fat high sugar diet without testosterone supplementation; CFD+T, control diet with testosterone supplementation; HFHS+T, high fat high sugar diet with testosterone supplementation; HFHS+Sham, high fat high sugar diet with sham surgical implantation. Female mouse groups **(D–F)**: CFD-no E2, control diet without 17β-estradiol supplementation; HFHS-no E2, high fat high sugar diet without 17β-estradiol supplementation; CFD+E2, control diet with 17β-estradiol supplementation; HFHS+E2, high fat high sugar diet with 17β-estradiol supplementation; HFHS+Sham, high fat high sugar diet with sham surgical implantation.

Body weight did not significantly influence any wheel-running measurement (e.g., distance—males: *p* = 0.64; females: *p* = 0.08; duration—males: *p* = 0.71; females: *p* = 0.28; or speed—males: *p* = 0.20; females: *p* = 0.21) and thus, was dropped in other analyses. The male HFHS mice (HFHS-no T) ran significantly less distance (*p* = 0.02; Figure 3A) and had lower duration of activity (*p* = 0.01; Figure 3B) when compared to control mice (CFD-no T), but speed remained unchanged (*p* = 0.77; Figure 3C). In female HFHS mice (HFHS-no E2), there was also a significant decrease in distance ran (*p* = 0.02; Figure 3D), but no alteration in duration (*p* = 0.06; Figure 3E), or speed of activity (*p* = 0.17; Figure 3F) compared to the control mice (CFD-no E2).

### Sex steroid assays

**Figure 4:**
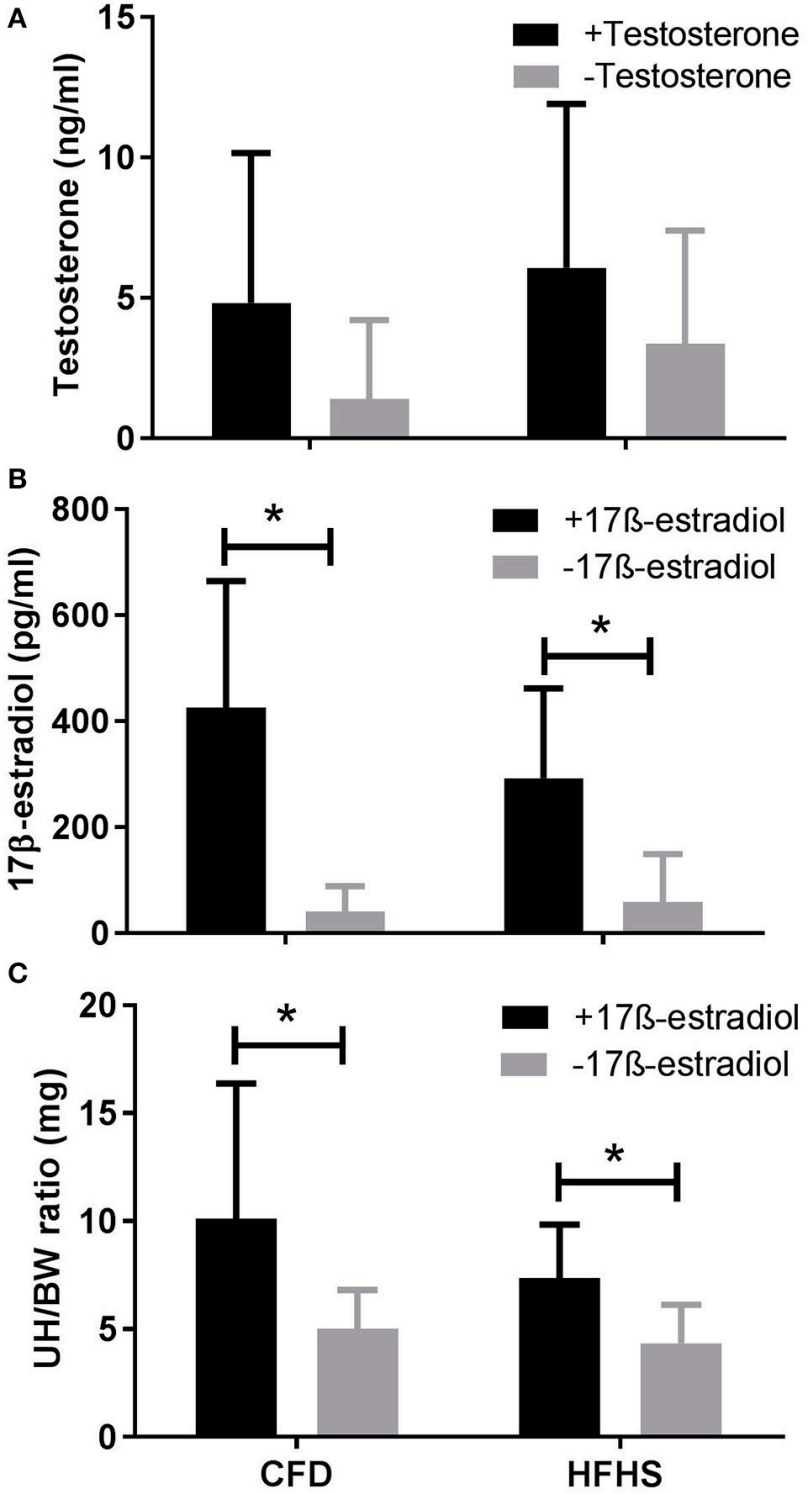
Sex hormone levels in CFD and HFHS male and female mice with- and without- supraphysiological sex hormone supplementation. CFD, control mice; HFHS, high fat/high sugar mice. **(A)** Serum levels of testosterone in male CFD and HFHS mice with- and without- testosterone supplementation. **(B)** Serum levels of 17β-estradiol in female CFD and HFHS mice with- and without 17β-estradiol supplementation. **(C)** Uterine horn/body weight ratio in female CFD and HFHS mice with- and without 17β-estradiol supplementation. UH/BW, Uterine horn/body weight. ^*^Significantly higher than non-supplemented levels of 17β-estradiol (*p* = 0.0003) and uterine horn/body ratio (*p* = 0.01). Testosterone supplementation did not significantly increase serum testosterone levels in male CFD or HFHS mice (*p* = 0.13).

In male mice, serum testosterone levels (ng/ml) in the CFD and HFHS were not significantly different when measured following 9 weeks of diet treatments (*p* = 0.27, Figure 4A). The average coefficient of variance between triplicates from each sample was 5.9% (±2.9). All sample values fell within range of the assay kit's range (0.1–25 ng/ml with a sensitivity of 0.066 ng/ml).

In female mice, serum 17β-estradiol concentration values were not significantly different between the HFHS and CFD mice (*p* = 0.60, Figure 4B). The average coefficient of variance between triplicates from each sample was 3.17% (±1.5). The 17β-estradiol assay was able to detect 20–2,000 pg/ml (sensitivity of 8.68 pg/ml) and the values for all of our analyzed samples fell within this range. Additionally, as a secondary confirmatory measure to the 17β-Estradiol values, we also calculated the ratio of uterine horn to total body weight in the female mice. We found no significant difference (*p* = 0.54) between the CFD and HFHS mice (Figure 4C) which supports the lack of difference in estrogen values between the diet groups (Figure 4B).

### Experiment 2—supraphysiological sex hormone supplementation and acute wheel-running

Given that the primary purpose of this experiment was to test whether sex hormone supplementation in male (testosterone) and female (17β-estradiol) would recover the HFHS-induced reductions to acute running-wheel activity, the Experiment 1 CFD mouse running-wheel activity served as the baseline running-wheel activity for male (CFD-no T) and female (CFD-no E2) mice, while the Experiment 1 HFHS group served as the baseline running-wheel activity of overfed male (HFHS-no T) and female (HFHS-no E2) mice.

In male mice supplemented with a silastic capsule containing testosterone (CFD+T and HFHS+T; Figure 4A), the serum levels of testosterone (ng/ml) in the CFD+T and HFHS+T mice were not different when compared to their control counterparts in experiment 1 (CFD-no T and HFHS-no T), whether comparing the effect of diet (*p* = 0.57) or testosterone supplementation (*p* = 0.22; Figure 4A). Similar to Experiment 1, the average coefficient of variance between triplicates/duplicates from each sample was 4.1% (±2.9) and all values fell within range of the assay kit. In female mice, the serum levels of 17β-estradiol (pg/ml) in the CFD+E2 and HFHS+E2 mice were higher than the control animals (*p* = 0.0003; Figure 4B), but there was not an effect of diet type (*p* = 0.42) on this response. The average coefficient of variance between triplicates/duplicates from each sample of was 3.2% (±1.4). Additionally, 17β-estradiol supplementation increased uterine horn weight (*p* = 0.01; Figure 4C), with no effect of diet type (*p* = 0.22), which supports our estrogen concentration values (Figure 4B).

When considering the effect of sex hormone supplementation, in the male mice, there was an overall decrease in distance ran (*p* = 0.01; Figure 3A) and duration of activity (*p* = 0.001; Figure 3B) in all HFHS mice as compared to the control mice, but no difference in speed of activity (*p* = 0.61; Figure 3C). The HFHS-no T and HFHS sham groups ran significantly less than their control counterparts (CFD-no T; *p* < 0.05; Figures 3A–C) confirming that HFHS reduced activity. Both the distance and duration activity in the HFHS+T were not different from the CFD-no T group (*p* > 0.05) or the HFHS-no T or HFHS-Sham groups (Figures 3A,B).

The female mice exhibited an overall decrease in distance (*p* = 0.002; Figure 3D) and duration (*p* = 0.003; Figure 3E), but not speed of activity (*p* = 0.08; Figure 3F) with HFHS with or without supplementation. A Tukey's *post-hoc* test indicated that all HFHS groups (HFHS sham, HFHS+E2, HFHS-no E2), in addition to the CFD+E2 group, ran significantly less distance than their control counterparts (CFD-no E2; *p* > 0.05; Figure 3D). For duration of activity, all HFHS female mice ran significantly less time than their control counterparts (CFD-no E2; *p* > 0.05; Figure 3E). Thus, estrogen supplementation did not alter the overfeeding-induced inhibition of activity in female mice.

### Experiment 3—2-week access to wheel-running

**Figure 5:**
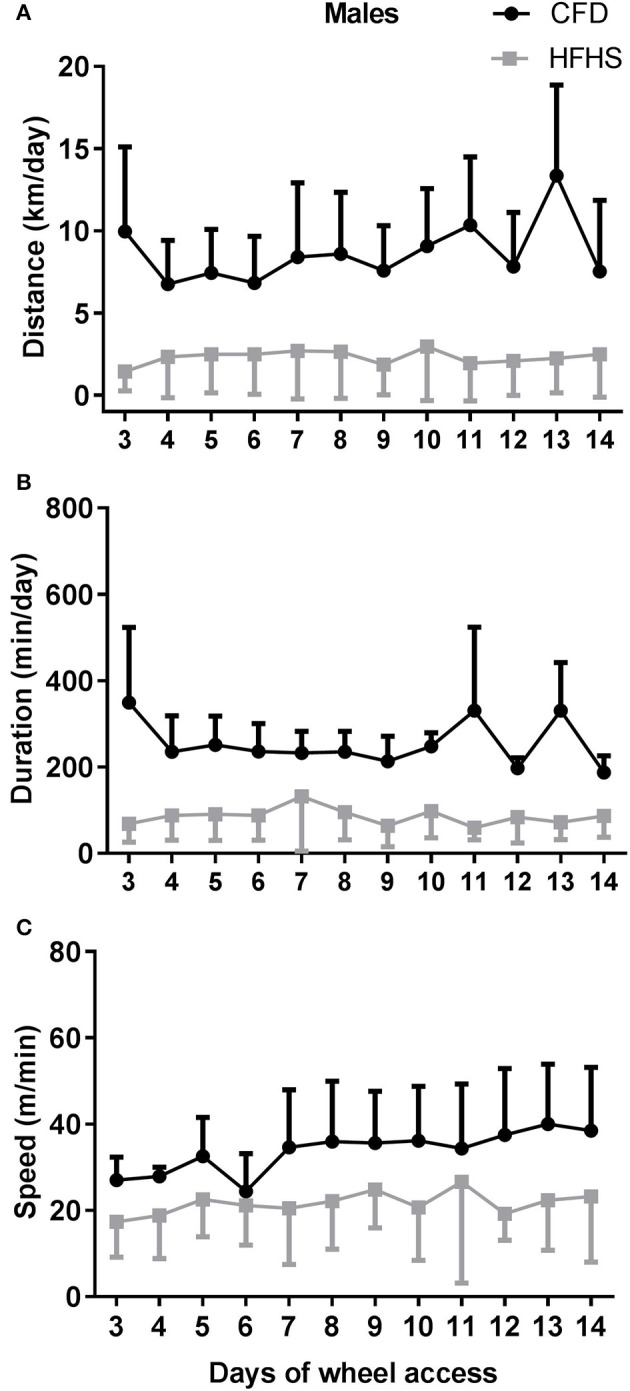
Two-week wheel running activity in CFD and HFHS male mice. **(A)** Male average daily wheel running distance (km/day). **(B)** Male average daily wheel running duration (min/day). **(C)** Male average daily wheel running speed (m/min). There were significant differences in the average daily distance (*p* = 0.03) ran over a 2-week time period of running-wheel access. There were no significant differences in the speed (*p* = 0.45), or duration of activity (*p* = 0.06).

**Figure 6:**
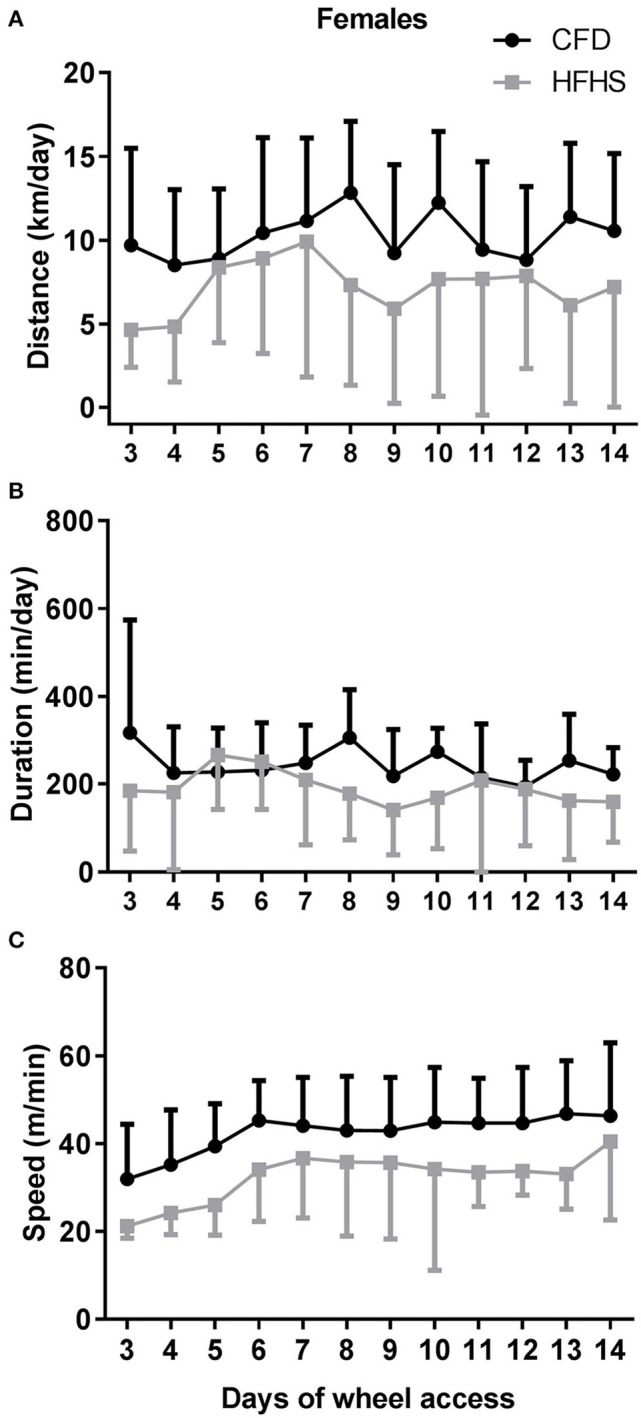
Two-week wheel running activity in CFD and HFHS female mice. **(A)** Female average daily wheel running distance (km/day). **(B)** Female average daily wheel running duration (min/day). **(C)** Female average daily wheel running speed (m/min). There were significant differences in the average daily distance (*p* = 0.02) ran over the 2-week time period of running-wheel access, with the decrease in distance due to decreased speed (*p* = 0.01), and not duration of activity (*p* = 0.49).

In general, the 2-week period of running-wheel access did not alter the HFHS-induced decreases in wheel running in either male (Figure 5) or female mice (Figure 6). The HFHS male mice compared to the CFD mice showed an overall significant decrease in distance (*p* = 0.01, Figure 5A), but not the speed (*p* = 0.45; Figure 5C) or duration (*p* = 0.06; Figure 5B) of activity. In the female HFHS mice (Figure 6), there was also a significant reduction in the daily distance run (*p* = 0.02; Figure 6A) and speed (*p* = 0.01; Figure 6C) with no change in duration (*p* = 0.49; Figure 6B).

In male mice, following 2-weeks of wheel access (Figure 1C), the overall significant difference in percent body fat (*p* < 0.0001) between the CFD and HFHS mice was due to diet type (*p* < 0.0001) and not due to running-wheel access (*p* = 0.30). Similarly, in female mice (Figure 1D) there was an overall significantly higher (*p* < 0.0001) percent body fat in the HFHS female mice due to diet type (*p* < 0.0001), but not due to running-wheel access (*p* = 0.07).

After 2 weeks of running, in male mice, there were no significant differences (*p* = 0.69) in serum testosterone concentration values between the CFD (3.79 ± 6.0 ng/ml) and HFHS (5.33 ± 5.6 ng/ml) mice with all values falling within range of the assay kit's assay range and the average coefficient of variance between triplicates from each sample was 3.3% (±1.5%). While we attempted to analyze 17β-Estradiol serum concentrations in the female mice in this experiment three times in total, the results we obtained for each of the ELISAs were undetectable (~50% undetectable), presumably due to very low estradiol concentrations in these mice. Thus, uterine horn weights were used as a surrogate measure for estrogenic status in the female mice for this aim. There was a significant difference (*p* = 0.02) between the CFD (3.9 mg ± 1.45) and HFHS (2.24 mg ± 0.34) mice that suggested lower circulating estrogen in the HFHS mice after 2 weeks of wheel running exposure.

To determine whether the decrease in activity with the HFHS diet seen in the experiment 1 results replicated we compared day 3 of running-wheel activity of the mice experiment 3 (2 weeks of wheel running) with the results from experiment 1. Similar to experiment 1, within experiment 3, we found significant acute reductions in distance ran for HFHS male (*p* = 0.01) and female (*p* = 0.001) mice; however, unlike in experiment 1, the reduction to duration in males (*p* = 0.01), and reduction to speed of activity in females (*p* = 0.01) was significant. When we compared the magnitude of the reduction in activity between experiment 1 and experiment 3 CFD and HFHS mice, there no significant differences in the magnitude of HFHS induced activity reduction in either male (distance, *p* = 0.29; duration, *p* = 0.35; speed, *p* = 0.15) or female mice (distance, *p* = 0.86; duration, *p* = 0.76; speed, *p* = 0.49). Thus, given that the same overall acute activity inhibition occurred with the HFHS diet in the two experiments indicates that the overfeeding-induced inhibition of activity was repeatable.

## Discussion

The primary purpose of this study was to investigate whether chronic overfeeding, with a HFHS diet, altered wheel running in mice and if changes in activity were associated with related alterations in sex hormones. The efficacy of the HFHS diet to induce overfeeding in mice was confirmed by the significantly greater daily calories consumed, and increases in total body weight and percent body fat, in both male and female mice fed the HFHS diet. From this, we were able to demonstrate that chronic overfeeding leads to significant reductions in acute running-wheel distance in both male (≈70%) and female mice (≈57%). This HFHS-induced reduction in acute activity was replicated to a similar magnitude in all three experiments. Surprisingly, however, chronic overfeeding did not alter sex hormone levels in either male or female mice, nor did supraphysiological sex hormone supplementation recover HFHS-induced reductions to activity in male or female mice. Thus, these observations suggest that the overfeeding-induced reduction in wheel running is independent of sex hormone involvement. Lastly, when we increased running-wheel exposure time to 2-weeks, the daily distance ran for male and female HFHS mice remained significantly lower than the CFD mice throughout the 2-week period suggesting that simply having access to a mode of exercise is not sufficient to overcome diet-induced reductions to activity. Thus, chronic overfeeding repeatedly inhibited daily wheel running; though, the mechanism underlying these behaviors still remains unclear.

### Effect of chronic overfeeding on acute running-wheel activity

Our findings of an overall decrease in activity with overfeeding are supported by human studies that have indirectly linked caloric excess with reductions in activity (Levine et al., 2008; Schmidt et al., 2012). Schmidt et al. (2012) showed that chronic overfeeding in otherwise healthy, obesity-prone men and women aged 25–35 years, resulted in significantly reduced spontaneous physical activity. Another study by Levine et al. (2008) demonstrated that daily walking distance was significantly decreased when lean and obese subjects (ages 39 ± 8 years) were overfed by 1,000 kcals/day for 8 weeks. Interestingly, Levine et al. speculated that the decrease in activity was due to a “decreased sensitivity to central neurotransmitters that drive walking” implying a potential dietary-mediation in the underlying mechanisms that drive physical activity.

In mice, several studies have studied wheel-running activity after exposure to various types of diets, such as, the specialized “Western diet” (Bjursell et al., 2008; Meek et al., 2010, 2014) and the “Very high fat diet” (Funkat et al., 2004), with several observing decreases in activity with overfeeding while one showed increased activity with overfeeding. Meek et al. (2010) examined the effect of a specialized “Western diet” (42% fat) on voluntary wheel running in mice selectively bred for high running-wheel activity for 52 generations, and found that the diet led to significant increases in running-wheel activity in mice selected for high running-wheel activity, but not the selected control line. In these mice selected for high wheel-running activity, Meek et al. (2010) speculated that the high fat diet stimulated further increases in their running activity, and not the selected controls, given these mice normally expend their available energy stores on a daily basis (because of their high activity status), and the extra fat provided fuel source to enable the mice to run further. In comparison to this current study, Meek et al. (2010) presented both the diets and running-wheels simultaneously (3–8 weeks of age), whereas in our study, the mice were overfed for a period of 9-weeks before having access to a running-wheel. Thus, because of the unique genetic make-up of the mice used by Meek et al. and the chronic nature of our animals' exposure to the HFHS diet, it is unclear if our observations can be appropriately compared to the responses of the Meek et al. (2010) study.

In addition to the indirect evidence from the previously listed human studies (Levine et al., 2008; Schmidt et al., 2012), three studies support our findings of a decrease in activity with overfeeding in mice. Funkat et al. (2004), using male C57BL/6J mice (the same strain as in the current study), demonstrated that providing a “very high fat diet” (60% fat) led to significant reductions (≈30% estimated) in acute running-wheel activity when activity was measured for 3 days following a 6-week period of being on the diet (8–14 weeks of age). A similar study by Bjursell et al. (2008) showed that C57BL/6J mice receiving a Western diet over a 21-day period, had significantly lower home-cage activity when compared to control counterparts. While we have shown that home-cage activity and wheel-running activity are not necessarily correlated in all strains of mice (Lightfoot et al., 2010), home-cage activity is considered a locomotor behavior and reductions in home-cage activity has been used as an indication of reductions in daily activity (Williams et al., 2014). Lastly, while the purpose of this study was not specifically to “overfeed” mice, a study by Rendeiro et al. (2015) provided isocaloric diets consisting of either 18% fructose or 18% glucose to male C57BL/6J mice for 11-weeks, and found significant reductions to home cage activity (≈20% reduction) in the 18% fructose fed mice. Thus, the study by Rendeiro et al. (2015) demonstrated a solitary effect of fructose on reduced activity independent of overfeeding. The earlier study by Funkat et al. (2004) showed a decrease in activity with the “very high fat diet”; however, given the mice were overfed by the diet in this study, it is unclear whether fat or increases in fatty acids have an effect on activity independent of overfeeding. Taken together, our findings, in addition to the findings from human (Levine et al., 2008; Schmidt et al., 2012) and rodent studies (Funkat et al., 2004; Bjursell et al., 2008; Rendeiro et al., 2015) suggest that chronic overfeeding and/or high diet composition of a particular nutrient such as, fructose (Rendeiro et al., 2015) can lead to significant detriments in physical activity.

With the findings of a decreased activity status due to overfeeding, we hypothesized that alterations in sex hormones were a potential causative mechanism for this inhibition. The primary sex hormones in males (testosterone) and females (17β-estradiol) have been shown to be potent biological regulators of wheel running in rodents (Richter and Wislocki, 1928; Roy and Wade, 1975; Bowen et al., 2012). Additionally, a separate body of literature suggesting that sex hormones are markedly altered by chronic overfeeding (e.g., Teerds et al., 2011; Bouchard et al., 2014) led us to hypothesize that if overfeeding altered activity levels, that this alteration was possibly mediated through a concurrent alteration in sex hormones.

To our surprise, we found that the serum levels of testosterone and 17β-estradiol were not significantly altered by the HFHS diet in male or female mice. As a confirmatory measure to determine whether the sex hormones were involved in mediating the HFHS-induced reduction in wheel running, we gave exogenous sex steroid hormones and then compared the acute running-wheel activity levels to control counterparts. In male mice testosterone supplementation did not significantly recover the diet-induced reduction in activity. Of important note is that the testosterone levels in the HFHS fed male mice were within normal ranges as reported in the literature (Larocca et al., 2011; Fan et al., 2015; control C57BL/6J mice) and similar to the testosterone levels of the control male mice of this study. Thus, given that levels of testosterone were not altered by the HFHS diet, and that testosterone supplementation did not significantly increase wheel running above the levels of the non-supplement HFHS or HFHS sham groups, suggests that overfeeding reductions to activity occurs independent of testosterone in males. In female mice, in spite of 17β-estradiol supplementation increasing circulating 17ß-estradiol levels, the supplementation had no effect on activity regulation in HFHS females as the activity (distance and duration) remained significantly lower than the activity in the control animals. Thus, in general, the sex hormones in this study did not appear to have mediated the inhibition of wheel running caused by overfeeding.

Given the prior work demonstrating an effect of overfeeding (Bouchard et al., 2014) and/or obesity (Teerds et al., 2011) on reduced testosterone in men, it is uncertain why our results in male mice do not demonstrate similar reductions in testosterone levels with a high fat high sugar diet; though, one possible explanation is due to variability in genetic background. In the study by Bouchard et al. (2014), twin male subjects were overfed by 1,000 kcals/day for 8 weeks (6 days/week), and found that serum levels of testosterone were only affected in subjects who had the greatest sensitivity to diet according to body composition changes following overfeeding (i.e., higher total body weight, percent body fat, and fat mass). Similarly, Sato et al. (2014), showed greater decreases in serum testosterone with overfeeding in men with a family history of type 2 diabetes when compared to men that did not have that history. Given that there are established differences in susceptibility to diet-induced changes to sex hormone levels due to genetics (Bouchard et al., 2014) and familial influences (Sato et al., 2014), it is possible that using a different strain of mice would have resulted in alterations in sex hormone levels with overfeeding. However, importantly, our results show that even without alterations in sex hormone levels, overfeeding still produced marked and significant decreases in daily activity in both sexes, suggesting that sex hormones are not involved as the primary mechanism for this effect.

Our conclusions regarding sex hormones must be considered cautiously given that determination of sex hormone levels from the small plasma samples in mice is generally considered a difficult task (Bowen et al., 2012). While the coefficient of variation for our duplicate/triplicate results for both testosterone and 17β-estradiol measurements were at or below 5%, the variability we observed between samples is indicative of the measurement difficulty. In one aspect, the variability in testosterone concentrations is not surprising given the wide range of testosterone values reported across the literature in adult control C57BL/6J male mice with normal values reported as low as 0.16 ± 0.9 ng/ml (Larocca et al., 2011) to upwards of 8.71 ± 2.07 ng/ml (Fan et al., 2015). The reason for the high variability across the literature is unclear, but could potentially be related to the different ELISA manufacturer kits and/or an effect of the time of day the samples (serum) was taken which generally has not been accounted for in studies (Kanková et al., 2011; Larocca et al., 2011; Fan et al., 2015). In the current study, we controlled for circadian state by taking all samples between the hours 9:00–11:00 am in the male mice, which is during their sleep period. Taken together, the testosterone values obtained in our study, while variable, did fall within normal ranges provided by previous studies (Kanková et al., 2011; Fan et al., 2015) of mice that were of similar age and strain. Similarly, in the female mice, we controlled for potential confounding variables by both circadian rhythms and the menstrual cycle with the ranges and variability of 17β-estradiol levels we observed being similar to what is noted in the literature (Bryzgalova et al., 2008; McLean et al., 2012). Thus, overall, our results suggest that neither testosterone nor 17β-estradiol hormone supplementation played a role in altering activity in the HFHS mice and suggest that overfeeding reduces activity independently of the sex hormones.

It has been suggested that weight alone may influence physical activity (Tucker et al., 2013; Laroche et al., 2015). In this study, regardless of the diet-type provided, we found that body weight had no effect on any measure of activity in the male or female mice. The insignificant influence of body weight on activity level in the mice of this current study was not surprising given prior work from our lab that have also shown an insignificant effect of body weight on wheel running in mice (Lightfoot et al., 2004, 2010; Leamy et al., 2009). One such study from our lab demonstrated this lack of influence of body weight on distance ran from data gathered from 41 inbred mouse strains (Lightfoot et al., 2010). Additionally, we have specifically tested the association between body weight and wheel running behaviors (Leamy et al., 2009) from an F_2_ generation of mice that were an intercross of two inbred lines that were found previously to have significantly different wheel running levels (Lightfoot et al., 2004). In these studies, we have found that the direct-effect quantitative trait locus (QTLs) for wheel running behaviors traits were largely independent from those for body weight (i.e., only one of seven QTLs associated with the physical activity phenotype co-localized with body weight). Thus, these studies suggested, on the basis of genetic background, that body weight is not a significant influencer of physical activity. Most recently, a study by Friend et al. (2016), provided additional evidence supporting the lack of influence of body weight on activity, where they found that obesity reduced activity in mice, but that restoration of dopamine 2 receptor binding of the basal ganglia which was altered by the diet, recovered baseline activity in spite of no change in body weight. In human populations, all of the available studies are cross-sectional in nature where activity is self-reported. The use of these studies to associate activity with body weight in complicated by the inability to control for the genetic regulation of activity, which has been shown in both humans (Stubbe et al., 2006) and animals (Lightfoot et al., 2010). Thus, our current understanding with regard to whether body weight influences activity is limited to the mouse model. Therefore, taken together our data demonstrates that providing a HFHS diet reduces wheel running repeatedly; and importantly, wheel-running activity is not associated with increases in body weight or with alterations in levels in the sex hormones.

### Effect of 2-week running-wheel access

It is accepted that repeated exposure to exercise has beneficial effects on a wide-variety of systems, including both metabolic and cardiovascular adaptations. Thus, we exposed our HFHS animals to 2-weeks of running-wheel exposure without changing their diets to determine if any of the overfeeding alterations (i.e., body composition, wheel running, and sex hormone levels) we observed would be reversed by repeated and increased exposure to exercise. However, even with repeated wheel-running exposure, the average daily distance for both HFHS male and female mice remained significantly lower compared to the CFD mice, suggesting that wheel exposure alone was not of sufficient impact to reverse the overfeeding-induced activity inhibition. Additionally, the activity exhibited by the HFHS mice over that 2-week period was insufficient to alter the body composition changes that resulted from overfeeding. It should be considered that the dietary treatments for the mice were not changed during this 2-week wheel running exposure, suggesting that as long as the dietary modification was in place, exercise could not overcome the negative physiological consequences of the diet. Interestingly, long-term exposure to the running wheel appeared to substantially increase caloric intake of the HFHS mice (by 32 and 55% in males and females, respectively), suggesting that this was a possible reason why the HFHS mice did not alter body composition or further increase activity with exposure to activity. Mechanistically, it is unclear what led to this apparent sexual dimorphism effect where wheel exposure appears to stimulate excessive eating behavior in female, and not male, HFHS fed mice, but presents an interesting topic for future research. The finding that having access to exercise modalities, such as, the running wheel for mice, does not necessarily prompt increased physical activity is indirectly supported by human experiments where objective measurements of daily activity remain unchanged following implementation of “built environments” that are geared to promote physical activity (e.g., sidewalks, parks, gyms, etc.; Ferdinand et al., 2012). From an evolutionary perspective, the increase in food availability in Westernized societies has been hypothesized to have removed the stimulus to search for food, and as such, may contribute to the low levels of activity present in modern society (Lightfoot, 2013).

## Limitations

One potential limitation in this study was that wheel running behavior was the only measure of voluntary physical activity used in this study. Thus, it is still unknown whether other measures of activity (e.g., general cage activity; climbing) in mice may have been altered with the HFHS feeding. Running wheel activity was chosen for the purpose of our investigation given it closely resembles intentional human voluntary exercise behaviors (Meijer and Robbers, 2014). A secondary limitation to this study is that there remain other potential ways through which the sex hormones could be involved in overfeeding-induced reductions on activity that should be considered in future research. In this current study, we only considered circulating serum-levels of the sex steroids as an indication for overfeeding-induced alterations to sex hormone regulation and function. While unknown from this current investigation, it is possible that androgen receptors, estrogen receptors, and/or enzymatic activities could have been impaired due to the HFHS diet, while circulating concentrations of the sex hormones remained unchanged. For example, a recent study in humans by Sato et al. (2014) demonstrated that following 28-days of overfeeding in males (1,000 kcals/day above energy needs), skeletal muscle expression of steroidogenic enzymes, 3β- hydroxysteroid dehydrogenase (HSD) and 17β-HSD were significantly reduced by overfeeding, even though serum testosterone levels were not altered. In another recent study, Xu et al. (2015) demonstrated that the estrogen receptor α, in the medial amygdala, meditates estrogenic activity to stimulate physical activity in both male and female mice. Additionally, separate work has further demonstrated a high fat diet suppresses the estrogen receptor α (Gorres et al., 2011; Metz et al., 2016). Therefore, while sex hormone levels in circulation did not appear to affect physical activity outcomes in our current study, sex hormones mediate their effects via receptor signaling. Thus, our hypothesis that changes in sex hormones with chronic overfeeding may play a role in overfeeding-induced activity suppression may be supported if the intervention modulated levels of sex hormone receptors. Future studies should measure such receptors in the central nervous system and peripheral tissues. Lastly, with our employed model of overfeeding, it is not possible to independently delineate the influence of overfeeding vs. the effect of the composition of the diet *per se*. To further investigate this issue, additional studies would be needed to determine the solitary and/or combined effects of each component of the diet on wheel-running activity.

## Conclusions

This study shows that chronic overfeeding with a high in fat and sugar diet significantly and repeatedly reduces daily wheel running in both male and female C57BL/6J mice. These observations support earlier indirect human studies (Levine et al., 2008; Schmidt et al., 2012) and thus, increased caloric intake should be considered inhibitory to normal daily physical activity. Associated with the overfeeding response, we found no alterations in sex hormone levels in either males or females that would mechanistically explain the decreases in daily activity. Additionally, even with exogenous sex hormone supplementation, activity levels did not increase when the animals were maintained on a HFHS diet. Further, extended exposure to exercise, without removing the animals from the HFHS diet did not recover activity levels or body weight/body composition. Thus, while the inhibition of activity with overfeeding is repeatable, the causative mechanism appears to be independent of sex hormone influence. As such, further studies are needed to investigate the role of other overfeeding-induced effects on the wheel running response in order to elucidate the mechanism linking overfeeding and reduced wheel running.

## Author contributions

All authors listed for this manuscript contributed substantially to each of the following: To the conception or design of the work; or the acquisition, analysis, or interpretation of data for the work (HLV, ACL, NRW, JZG, and JTL); Drafting the work or revising it critically for important intellectual content (HLV, ACL, NRW, JZG, and JTL); and Final approval of the version to be published (HLV, ACL, NRW, JZG, and JTL). All authors are also in agreement to be accountable for all aspects of the work in ensuring that questions related to the accuracy or integrity of any part of the work are appropriately investigated and resolved (HLV, ACL, NRW, JZG, and JTL).

### Conflict of interest statement

The authors declare that the research was conducted in the absence of any commercial or financial relationships that could be construed as a potential conflict of interest.
